# Increased Brain Activation for Dual Tasking with 70-Days Head-Down Bed Rest

**DOI:** 10.3389/fnsys.2016.00071

**Published:** 2016-08-23

**Authors:** Peng Yuan, Vincent Koppelmans, Patricia A. Reuter-Lorenz, Yiri E. De Dios, Nichole E. Gadd, Scott J. Wood, Roy Riascos, Igor S. Kofman, Jacob J. Bloomberg, Ajitkumar P. Mulavara, Rachael D. Seidler

**Affiliations:** ^1^School of Kinesiology, University of MichiganAnn Arbor, MI, USA; ^2^Department of Psychology, University of MichiganAnn Arbor, MI, USA; ^3^Wyle Science, Technology and Engineering GroupHouston, TX, USA; ^4^Department of Psychology, Azusa Pacific UniversityAzusa, CA, USA; ^5^The University of Texas Health Science CenterHouston, TX, USA; ^6^NASA Johnson Space CenterHouston, TX, USA; ^7^Universities Space Research AssociationHouston, TX, USA; ^8^Neuroscience Program, Medical School, University of MichiganAnn Arbor, MI, USA

**Keywords:** dual task, head-down bed rest, fMRI, microgravity analog, brain activity

## Abstract

Head-down tilt bed rest (HDBR) has been used as a spaceflight analog to simulate the effects of microgravity exposure on human physiology, sensorimotor function, and cognition on Earth. Previous studies have reported that concurrent performance of motor and cognitive tasks can be impaired during space missions. Understanding the consequences of HDBR for neural control of dual tasking may possibly provide insight into neural efficiency during spaceflight. In the current study, we evaluated how dual task performance and the underlying brain activation changed as a function of HDBR. Eighteen healthy men participated in this study. They remained continuously in the 6° head-down tilt position for 70 days. Functional MRI for bimanual finger tapping was acquired during both single task and dual task conditions, and repeated at 7 time points pre-, during- and post-HDBR. Another 12 healthy males participated as controls who did not undergo HDBR. A widely distributed network involving the frontal, parietal, cingulate, temporal, and occipital cortices exhibited increased activation for dual tasking and increased activation differences between dual and single task conditions during HDBR relative to pre- or post-HDBR. This HDBR-related brain activation increase for dual tasking implies that more neurocognitive control is needed for dual task execution during HDBR compared to pre- and post-HDBR. We observed a positive correlation between pre-to-post HDBR changes in dual-task cost of reaction time and pre-to-post HDBR change in dual-task cost of brain activation in several cerebral and cerebellar regions. These findings could be predictive of changes in dual task processing during spaceflight.

## Introduction

It has been over half a century since the first man traveled in space. Since then, research has revealed a number of physiological and behavioral changes that are associated with spaceflight, such as alterations in muscle, bone, balance, mobility, cardiovascular function, and cognitive performance (Nicogossian et al., 1994; Manzey and Lorenz, 1998; Buckey, 2006; Mulavara et al., 2010; Strangman et al., 2014). Understanding the consequences of space travel could lead to countermeasures to mitigate spaceflight-related physical and psychological declines, resulting in improved mission performance.

Several ground-based models have been developed to simulate the spaceflight environment. Each of the analog environments imitates certain specific characteristics of spaceflight. For example, dry immersion is used as an analog of supportlessness (Pavy-Le Traon et al., 2007; Navasiolava et al., 2011); chronically elevated carbon dioxide is employed to simulate elevated CO_2_ levels in a sealed cabin (Wenzel et al., 1998); and isolated environments such as Antarctic station winter overs are used to mimic the limited social environment of prolonged spaceflight (Lugg, 2005; Basner et al., 2013). Particularly, head-down bed rest (HDBR) has been extensively utilized as an analog to study the effects of cephalic fluid shifts, foot sole unloading, and sensorimotor adaptation that can influence human physiology during exposure to microgravity. As a consequence of being in a head-down tilt supine position for a prolonged period, intravascular and extravascular fluids are shifted to the upper body, and head, as is observed in microgravity (Caprihan et al., 1999; Pavy-Le Traon et al., 2007). HDBR is also associated with foot sole unloading. Studies using the hindlimb unloaded rat model have documented changes in artery morphology and neural activity. For example, hindlimb unloading results in increases in the cross-sectional area and thickness of the basilar artery (Wilkerson et al., 1999), and attenuates baroreflex-mediated increases in sympathetic activity (Foley et al., 2005). In addition to fluid shifts and unloading, both spaceflight and HDBR conditions involve a process of adaptation to a new environment, which is associated with sensory reweighting and potentially neuroplastic changes. A series of head-down tilt degrees has been tested, and −6° was considered a good balance of acceptability and effect size on physiological changes (At'kov and Bednenko, 1992). Hence, most bed rest studies use head-down tilt of −6° to mimic cephalic fluid shifts in addition to axial body unloading.

People often must perform two or more tasks at the same time, e.g., walking while watching traffic lights, talking, eating, etc. Performance in one or both tasks typically declines when two tasks are carried out simultaneously (Strayer et al., 2003), especially when the tasks are not automated. For example, a meta-analysis documented decline of gait performance when a secondary cognitive task was conducted concurrently with walking (Al-Yahya et al., 2011). Such performance declines from single to dual task conditions are typically referred to as dual-task costs. Dual-task cost is thought to reflect the capacity for central processing (Tombu and Jolicoeur, 2003), with lower dual-task costs indicating a larger central capacity, or better cognitive ability. For example, the magnitude of dual-task cost is greater in older adults than in young adults (Verhaeghen et al., 2003; Riby et al., 2004). Dual-task declines also depend on the nature of the task. Motor tasks and tasks requiring substantial control exhibit larger dual-task costs than relatively simpler tasks or tasks that depend on automated processing (Riby et al., 2004; Al-Yahya et al., 2011).

Estimating the influence of spaceflight on dual task performance is important for understanding the constraints on future missions. According to the existing literature, the influence of spaceflight on dual task performance yields mixed results. For example, a single-case study reported impairments in dual task performance of concurrent unstable tracking and memory search during 8 days of spaceflight (Manzey et al., 1995). Another study of simultaneous unstable tracking and a reaction time task reported increased tracking error under dual task conditions during a 5- to 6-month space mission (Bock et al., 2010). In addition to dual task impairments, increased dual-task costs have also been documented. For example, Bock et al. found increased dual-task costs for tracking error during spaceflight (Bock et al., 2010) while a single-subject study also reported increased dual-task cost at the beginning of a space mission compared to pre-flight levels (Manzey et al., 1998). In contrast, a study of a 16-day space mission reported no performance impairments in either simultaneous tracking or manual reaction time performance (Fowler et al., 2000). Studies that have observed increased dual task costs with spaceflight attribute them to stress, fatigue, and sensorimotor adaptation (Manzey and Lorenz, 1998; Fowler et al., 2000; Bock et al., 2010). Although not all studies found a significant influence of spaceflight on dual tasking performance, the effects seen with the increased tracking error and longer reaction time persisted through the duration of spaceflight and even a few days post-spaceflight, raising the importance of effective dual tasking, especially during landing operations.

Dual task studies in spaceflight analog settings are scant. A 45-day HDBR study which examined time-based prospective memory with an ongoing word recall task identified impaired prospective memory during HDBR compared to pre-HDBR (Chen et al., 2013). Nevertheless, no impairment of dual tasking was found during another 17-day HDBR study, which used simultaneous memory search and unstable tracking (Shehab et al., 1998).

The discrepancy of dual task impairments during spaceflight and ground-based analogs could result from the heterogeneity of experiment settings. It is possible that some tasks are more sensitive to space-related changes than other tasks. Furthermore, previous studies had relatively small sample sizes; thus it is also possible that in some studies low statistical power led to null results.

Investigators have used functional neuroimaging to understand the neural basis of dual-task interference. Even when behavioral dual task costs are not evident, additional brain networks may be recruited to meet added demand, resulting in reduced neural efficiency. Although it is suggested that the activated brain regions under dual task conditions depend on the component tasks (Adcock et al., 2000), the prefrontal cortex has been identified as a key region engaged during dual tasking (D'Esposito et al., 1995; Szameitat et al., 2002). Greater activation in the left inferior frontal junction is found for dual task than for single task performance, and this effect decreases with training, implying an association between brain activity and dual tasking ability (Erickson et al., 2007; Dux et al., 2009). In addition to the prefrontal cortex, the parietal cortex, dorsal pre-motor cortex, and anterior cingulate cortex have also been proposed as putative components of a dual task processing network (D'Esposito et al., 1995; Szameitat et al., 2002; Marois et al., 2006).

Understanding the consequences of HDBR for dual tasking neural activity can provide insight into the influence of spaceflight on cognitive control and neural efficiency, and help to understand the factors affecting adaptation to microgravity. To the best of our knowledge, there are no published studies of the effects of spaceflight or analog environments on dual tasking neural activity. In the current study, subjects simultaneously performed target counting and bimanual button pressing tasks while fMRI data were acquired before, during and after a 70-day HDBR exposure; a protocol paper for this study has been published previously (Koppelmans et al., 2013). We chose to combine a motor and cognitive task because this combination has been investigated previously in spaceflight studies (Manzey et al., 1995; Fowler et al., 2000; Bock et al., 2010). We hypothesized that when compared to the pre-HDBR baseline and in comparison to a control group of subjects not undergoing HDBR: (1) dual task performance would deteriorate during HDBR; and (2) brain activity for dual tasking during HDBR would increase in prefrontal, parietal, pre-motor, and anterior cingulate cortices. We further predicted that activation changes from pre-to-post HDBR in frontal, parietal and cingulate regions would correlate with dual task performance changes.

## Methods

### Participants

Eighteen healthy male subjects participated in the 70-day, 6°-HDBR campaign. They were right-handed and aged 31.1 ± 4.7 years at the time of admission (range: 25.7–39.8 years). These subjects were enrolled 13–23 days before the start of HDBR and released 14 days after HDBR. During the 70-days of bed rest, participants remained in the head-down tilt position all the time except for 30 min at each meal, when they were allowed to support their head with their hand. The study took place in a bed rest facility at the University of Texas Medical Branch, Galveston TX. All subjects received financial compensation for their participation. Thirteen of the HDBR subjects participated in an exercise protocol (Ploutz-Snyder et al., 2014; Koppelmans et al., 2015), which started 20 days before HDBR and continued until the end of HDBR, 6 days per week. The other five HDBR subjects did not exercise. In the current study, all HDBR participants' data were analyzed as one group and exercise was not included as a factor because the majority of HDBR subjects exercised.

Another 12 healthy males participated as control participants not undergoing HDBR who were recruited by the Human Test subject facility at NASA-JSC. These control participants were leading their usual everyday life. These ground-based control subjects were aged 41.4 ± 9.9 years at the time of admission (range: 26.2–59.7 years). All the HDBR and control subjects passed an Air Force Class III equivalent physical examination. Both the HDBR study and control study were conducted in accordance with the declaration of Helsinki, and were approved by the institutional review boards of the University of Michigan, the University of Texas—Medical Branch (UTMB), and NASA-JSC. Written informed consent was obtained from all the participants.

### Cognitive test

**Figure 1:**
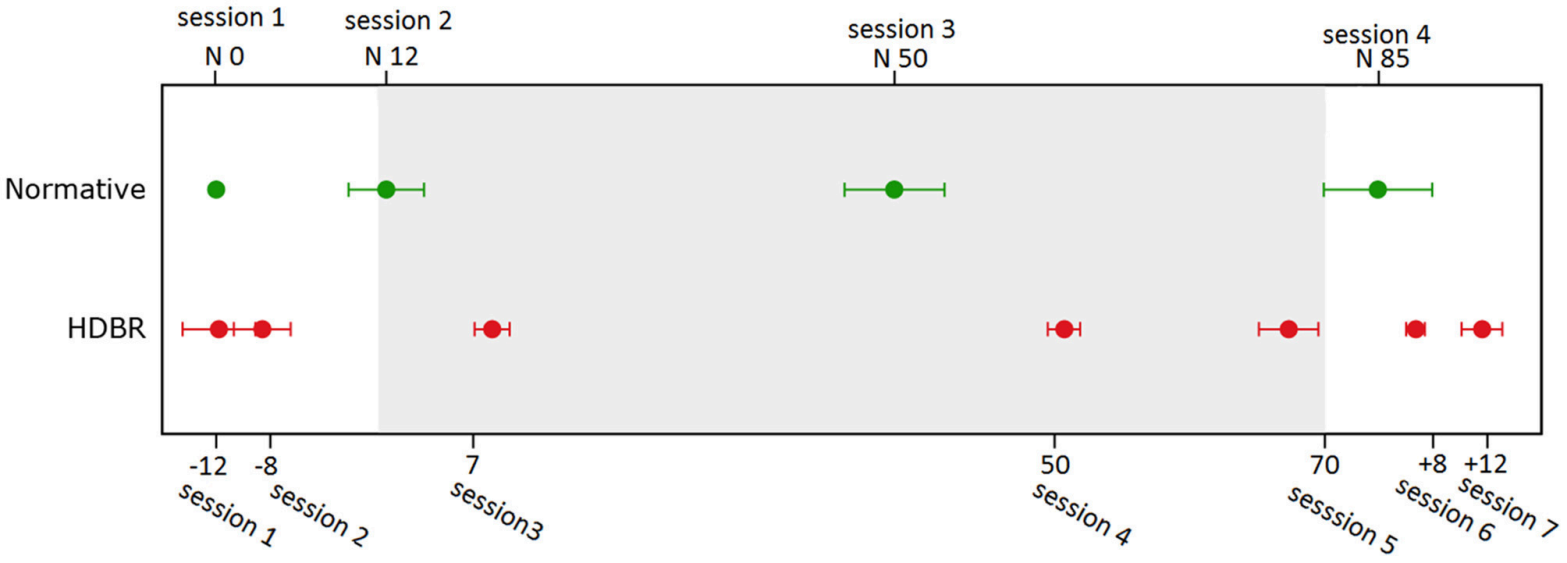
**Testing timeline for bed rest subjects and normative control subjects**. Error bars represent standard errors.

For HDBR subjects, testing was performed at 7 sessions: 14.1 ± 3.8 days and 7.9 ± 2.0 days before the start of HDBR, 8.4 ± 1.0 days, 50.6 ± 0.9 days, and 66.8 ± 1.8 days after the onset of HDBR, as well as 6.7 ± 0.8 days and 11.4 ± 1.6 days after HDBR. For control subjects, the task was repeated four times at days 0, 12.6 ± 9.7, 50.2 ± 12.8, and 84.8 ± 14.0. The timelines and deviations are presented in Figure 1.

**Figure 2:**
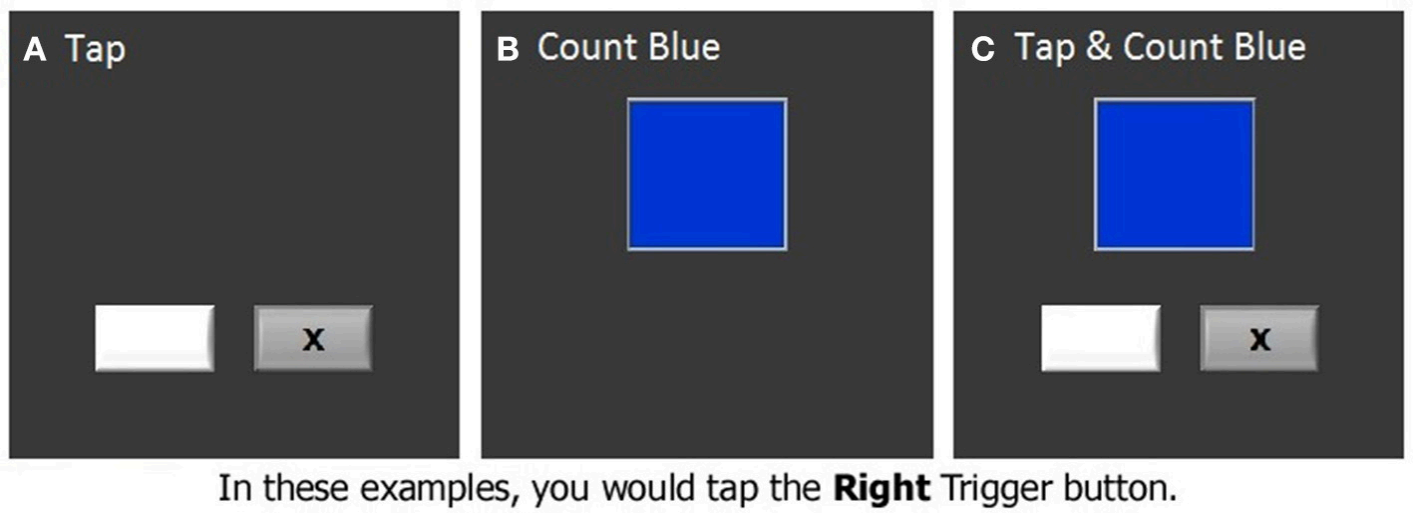
**The dual task test**. **(A)** Finger tapping task, **(B)** Counting task, **(C)** Dual task.

The finger tapping task was administered under both single and dual task conditions while we acquired fMRI data. In the finger tapping task, two stimulus boxes were presented on the screen. An “x” appeared in one of the two stimulus boxes with an inter-stimulus interval of 800 ms (Figure 2A). The stimuli were presented randomly, and subjects were instructed to indicate in which one of the stimulus boxes the “x” appeared by pressing the matching response button with either the right or the left index finger. In a secondary counting task, another stimulus box was presented, which changed color at a rate of 3 Hz (Figure 2B). Subjects were required to count the number of times that a target color appeared. The incidence of the target color was kept low (1–3%) so that subjects had to remain vigilant. In some blocks, which were referred to as dual task blocks, subjects were asked to perform the tapping and counting tasks simultaneously (dual task condition, Figure 2C, Seidler et al., 2002). For the dual task condition the box in which the colors were presented was centered and above the two boxes in which the “x” appeared. In the other blocks, subjects performed the tapping or counting task in isolation (tapping blocks and counting blocks, respectively). Two tapping blocks, two counting blocks, and two dual task blocks were included in an fMRI run, presented in a pseudorandom order. Each block was preceded and followed by 20-s rest periods, constituting an fMRI run of 260 s. There were two fMRI runs in each session. The latency of tapping timed from the onset of the “x,” as well as percent correct of tapping and counting were recorded and analyzed as dependent measures of performance.

### Image acquisition and processing

The fMRI scans for HDBR subjects were acquired on a 3-Tesla Siemens Magnetom Skyra MRI scanner, and the fMRI for control subjects were acquired on a 3-Tesla Siemens Magnetom Verio MRI scanner. The fMRI scan protocol was identical for all subjects. For the fMRI, a gradient echo T2^*^-weighted echo-planar imaging (EPI) sequence was used to collect the scans. The following parameters were used: *TR* = 3660 ms, *TE* = 39 ms, flip angle = 90°, FOV = 240 × 240 mm, slice thickness = 4 mm, slice gap = 1 mm, matrix = 94 × 94, voxel size = 2.55 × 2.55 × 5.0 mm, 36 axial slices. A T1-weighted gradient-echo pulse sequence was also collected for all subjects. For HDBR subjects, we used the following parameters: *TR* = 1900 ms, *TE* = 2.49 ms, flip angle = 9°, FOV = 270 × 270 mm, slice thickness = 0.9 mm; matrix = 288 × 288, voxel size = 0.94 × 0.94 mm, 192 slices; duration = ~4 min. For the control subjects, T1 images were collected with the following parameters: *TR* = 1900 ms, *TE* = 2.32 ms, flip angle = 9°, FOV = 250 × 250 mm, slice thickness = 0.9 mm, 192 slices; matrix = 512 × 512, voxel size = 0.49 × 0.49 mm, 3D T1 axial overlay; duration = ~4 min.

The functional images were corrected for slice timing and realigned to correct for head motion. The Artifact Detection Tool (ART, http://www.nitrc.org/projects/artifact_detect/) was used to quantify head motion and detect motion and global brain activation outliers, which were used as nuisance variables in the first level analyses. Then the images were normalized to the Montreal Neurological Institute (MNI) 152 space (Friston et al., 1995) using multiple steps: First, the T1 images were corrected for field inhomogeneity using N4ITK within an intracranial mask obtained from FSL's brain extraction tool (BET; Tustison et al., 2010). Second, the field bias corrected image was skull stripped using FSL's BET, with robust brain center estimation and a fractional intensity threshold of 0.1. Third, the skull stripped T1 images were co-registered to the mean fMRI EPI using SPM8. Fourth, advanced normalization tools (ANTs) were used to normalize the co-registered images to MNI152 common space (Avants et al., 2011). Then the warping parameters were applied for spatial smoothing with an 8-mm full-width half-maximum 3-dimensional Gaussian kernel. In addition to the whole brain normalization, a spatially unbiased atlas template of the cerebellum and brainstem (SUIT; Diedrichsen, 2006) was used for cerebellar normalization. fMRI runs with large head motion (>3 mm) were omitted.

The functional data were analyzed using SPM8. In the first level analysis, we calculated brain activity for each participant on a voxel-by-voxel basis for: tapping under the single task condition versus rest, the dual task condition versus rest, as well as dual task cost of brain activation, which is the additional brain activity under dual tasking above and beyond that observed in the single task condition.

**Figure 3:**
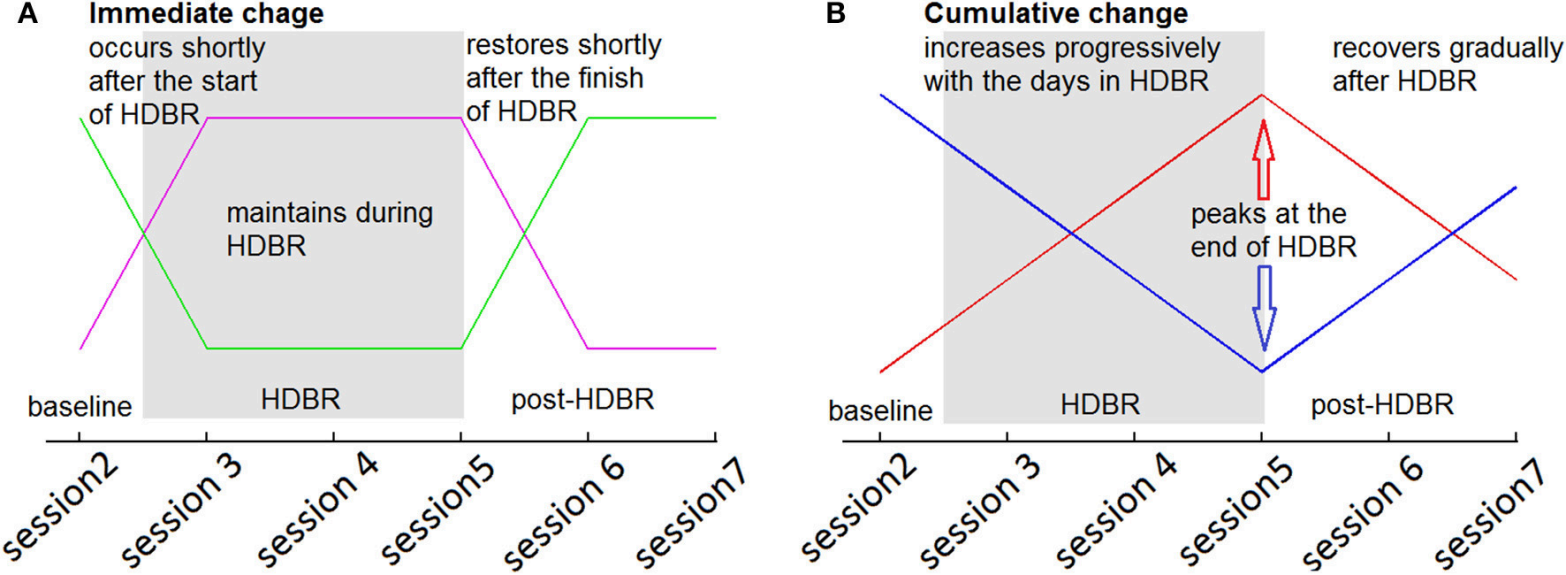
**Hypothesized HDBR-related brain activation changes. (A)** Immediate increase (violet) and immediate decrease (green). **(B)** Cumulative increase (red) and cumulative decrease (blue).

In the second level analyses, flexible factorial (SPM's mixed model equivalent) analysis was used to determine brain activity changes across sessions. We set several contrast vectors to test the hypothesized relative level of brain activation in each session. For the HDBR subjects, we sought to identify brain areas with two types of HDBR-related activation changes: immediate change and cumulative change. The immediate change was assumed to be sensitive to HDBR status, and was hypothesized to take place shortly after the start of HDBR, to be maintained during HDBR, and to restore shortly after the finish of HDBR (Figure 3A). The cumulative change was presumed to increase progressively with the days in HDBR, peaking at the end of HDBR, and recovering gradually after HDBR (Figure 3B). The first measurement session was regarded as a practice session, thus the fMRI data in the first session was excluded. For HDBR subjects, the activation levels in the later six sessions (2nd to 7th) were evaluated using contrast vectors as weights. For the control subjects, the first session was also regarded as practice. Therefore, we estimated linear increases and decreases in brain activation across the 2nd, 3rd, and 4th sessions for control subjects to evaluate any further practice effects.

In the second level analyses for HDBR subjects, we also calculated the brain activation difference between the baseline (2nd session, 7.9 ± 2.0 days before HDBR) and the end of HDBR (5th session, 66.8 ± 1.8 days in HDBR), in order to identify brain regions in which the activation change was significantly associated with behavioral change. For all fMRI analyses, the alpha level was set at 0.001 (uncorrected for multiple comparisons), and the extent threshold was 10 voxels.

### Statistical analyses for behavioral data

Linear mixed model analysis was used to compare the time courses of behavioral change in HDBR versus control subjects. Subject number was entered as a random variable. Time was entered as the number of days from the second session, which was treated as a continuous variable. The potential age effect on behavior was controlled statistically. For HDBR subjects, data from the post-HDBR sessions were excluded so we could examine whether the slope of change within bed rest differed from any potential practice effects in the control subjects. For all subjects, the data in the first session was dropped as a practice. Thus the behavioral data in the 2nd, 3rd, 4th, and 5th sessions (baseline and during HDBR) for HDBR subjects and behavioral scores in the 2nd, 3rd, and 4th sessions for control subjects were included. Restricted maximum likelihood (REML) was used. We used Stata 14 to analyze the behavioral data, and set the alpha level at 0.05. Bonferroni correction was used to correct for multiple comparisons.

## Results

### Behavioral results

**Figure 4:**
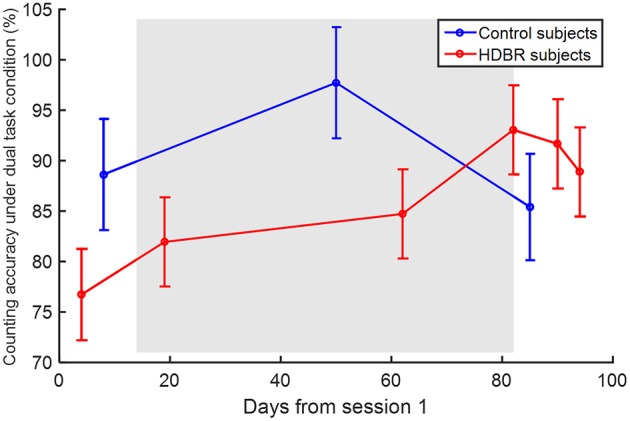
**Counting accuracy under the dual task condition**. Error bars represent the standard errors.

There was no effect of age on any of the behavioral scores, so it was not considered in any additional analyses. HDBR subjects showed a lower counting accuracy under the dual task condition (β = −17.807, *p* = 0.038) than control subjects. There was also a significant interaction of group × time (β = 0.315, *p* = 0.048), indicating that the HDBR subjects showed greater improvement in counting accuracy under the dual task condition than the control subjects. The counting accuracies are illustrated in Figure 4.

### fMRI results

#### Brain activation for dual task at baseline

**Figure 5:**
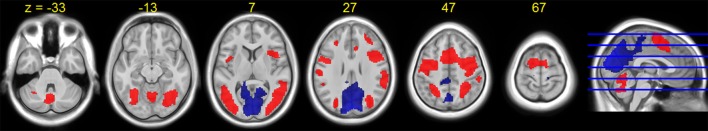
**HDBR group T-statistic maps for brain activation (red) and deactivation (blue) for dual task at baseline (session 2)**. Thresholded at abs (T) > 3.218.

The average HDBR group brain activation and deactivation for dual tasking at session 2 is shown in Figure 5. Consistent with the literature, we observed a distributed network involving the frontal, parietal, occipital, temporal, and cerebellar regions activated while subjects performed the dual task.

#### Brain activation change for tapping under single task condition

**Figure 6:**
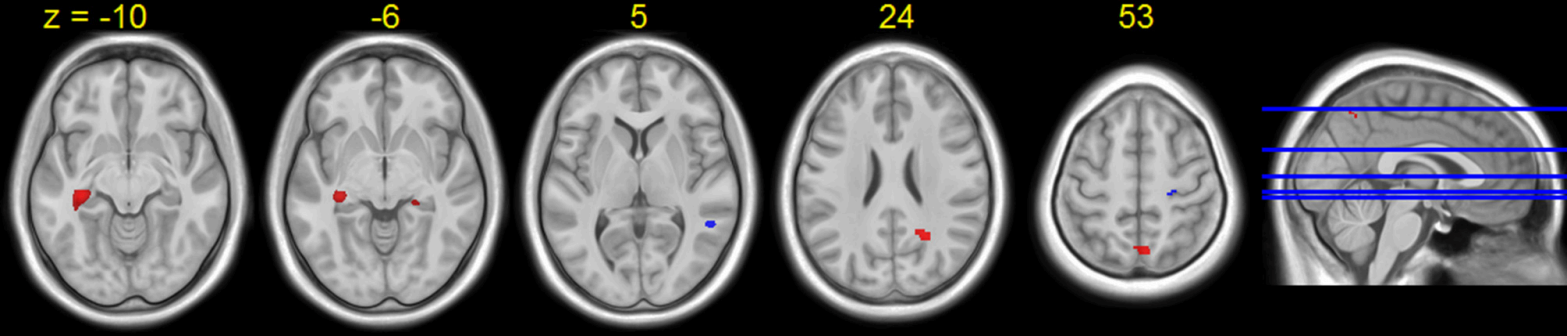
**Regions showing HDBR-related cumulative activation change for tapping under the single task condition**. Red: HDBR-related increase; blue: HDBR-related decrease. Group T-statistic map, thresholded at abs (T) > 3.218.

A few brain areas exhibited significant HDBR-related cumulative increases or decreases in activation for tapping under the single task condition (Figure 6, Table 1). However, no regions exhibited significant immediate change.

**Table 1:** **Regions showing HDBR-related cumulative activation changes for tapping under the single task condition**.

	**Region**		***k***	**Peak *T***	**Peak *p***	***x,y,z* (mm)**		
Increase	Parietal	R Precuneus	36	3.732	2E-04	24	−56	24
		R Precuneus	55	3.609	3E-04	6	−68	52
	Temporal	L Hippocampus	996	5.090	1E-06	−33	−26	−10
		R Parahippocampal Gyrus	69	3.432	5E-04	22	−32	−6
Decrease	Frontal	R Precentral Gyrus	10	3.380	5E-04	22	−26	54
	Temporal	R Middle Temporal Gyrus	125	3.531	3E-04	51	−47	5

k, cluster size; peak T, t-value at the peak voxel; peak p, p-value at the peak voxel; x, y, z, MNI coordinates of the peak voxel.

For the control subjects, several brain regions exhibited increases or decreases in activation for tapping under the single task condition from the 2nd session to the 4th session (Table 2), but nevertheless these regions did not overlap with those showing HDBR-related cumulative changes.

**Table 2:** **Regions showing activation changes for tapping under the single task condition in control subjects from sessions 2 to 4**.

	**Region**		***k***	**Peak *T***	**Peak *p***	***x,y,z* (mm)**		
Increase	Parietal	L precuneus	479	5.095	6E-06	−12	−54	12
	Cingulate	L Middle Cingulate Gyrus	31	3.784	3E-04	−4	−32	38
	Cerebellum	L Cerebellum_Lobule VIII	56	3.568	5E-04	−25	−51	−39
Decrease	Frontal	R Superiour Frontal Gyrus	10	3.609	5E-04	16	6	54
	Temporal	R Hippocampus	114	4.022	2E-04	37	−25	−6

k, cluster size; peak T, t-value at the peak voxel; peak p, p-value at the peak voxel; x, y, z, MNI coordinates of the peak voxel.

#### Brain activation change for tapping under dual task condition

**Figure 7:**
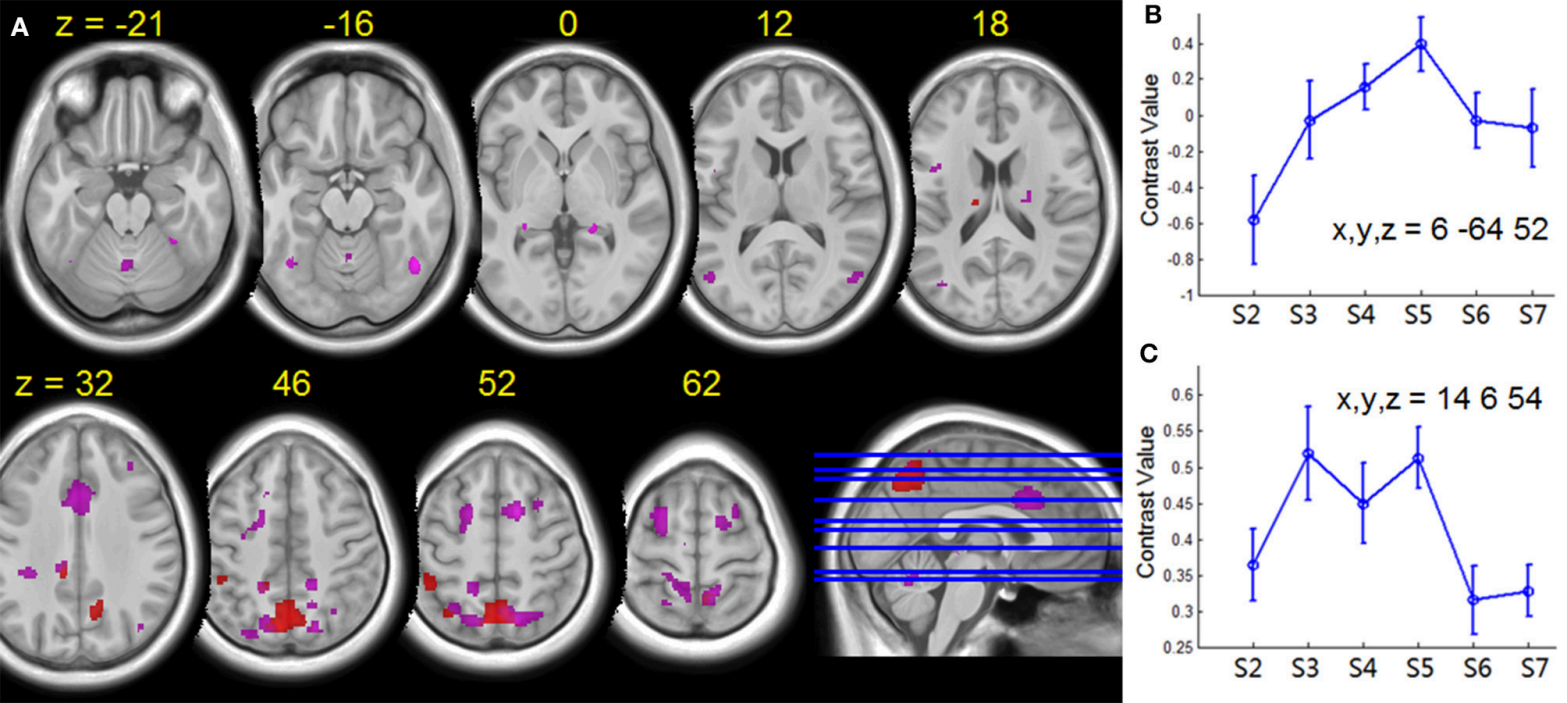
**(A)** Group T-statistic maps for the regions showing HDBR-related changes in activation for dual tasking, thresholded at abs (T) > 3.218. Red: HDBR-related cumulative increases; violet: HDBR-related immediate increases. **(B)** Example of cumulative increase across sessions. **(C)** Example of immediate increase across sessions. The MNI coordinates of the sample regions are indicated in **(B,C)**. Error bars represent standard errors across all the HDBR subjects.

For tapping under the dual task condition, a few regions in the parietal lobe and thalamus exhibited HDBR-related cumulative increases in activation (Figure 7, Table 3). The brain regions with immediate HDBR-related increases were distributed in the frontal, parietal, occipital, and temporal lobes, as well as cerebellum and subcortical regions (Figure 7, Table 4). However, no HDBR-related immediate decrease or cumulative decrease was found. There was no significant activation difference between the baseline (2nd session) and the last post-HDBR session (7th session) in the brain areas showing HDBR-associated activity change.

**Table 3:** **Regions showing HDBR-related cumulative increases in activation for dual tasking**.

**Region**		***k***	**Peak *T***	**Peak *p***	***x,y,z* (mm)**		
Parietal	R Precuneus	1042	4.834	3E-06	6	−64	52
	L Inferior Parietal Lobule	86	3.846	1E-04	−44	−42	50
	L Precuneus	33	3.669	2E-04	−16	−46	46
	L Superior Parietal Lobule	28	3.626	2E-04	−32	−64	54
	L Precuneus	10	3.287	7E-04	−30	−76	42
Cingulate	L Posterior Cingulate Gyrus	36	3.876	1E-04	−10	−34	32
Thalamus	L Thalamus	10	3.588	3E-04	−12	−18	18

k, cluster size; peak T, t-value at the peak voxel; peak p, p-value at the peak voxel; x, y, z, MNI coordinates of the peak voxel.

**Table 4:** **Regions showing HDBR-related immediate increases in activation for dual tasking**.

**Region**		***k***	**Peak *T***	**Peak *p***	***x,y,z* (mm)**		
Frontal	R Supplementary Motor Cortex	369	5.539	2E-07	14	6	54
	L Superior Frontal Gyrus	378	4.466	1E-05	−24	4	62
	L Operculum	24	3.629	2E-04	−44	2	16
	R Middle Frontal Gyrus	43	3.611	3E-04	36	36	34
	L Superior Frontal Gyrus	21	3.445	4E-04	−16	14	40
	L Supplementary Motor Cortex	10	3.428	5E-04	−8	−16	58
Parietal	R Precuneus	987	4.393	2E-05	14	−68	52
	R Precuneus	57	4.174	4E-05	16	−44	46
	L Inferior Parietal White Matter	59	3.711	2E-04	−32	−36	32
	R Precuneus	17	3.469	4E-04	16	−56	46
Cingulate	R Anterior Cingulate Gyrus	405	4.299	2E-05	2	16	30
	L Posterior Cingulate Gyrus	24	3.879	1E-04	−12	−32	32
Occipital	L Middle Occipital Gyrus	11	3.521	3E-04	−20	−88	4
	L Middle Occipital Gyrus	28	3.420	5E-04	−34	−74	22
	R Middle Occipital Gyrus	11	3.306	7E-04	40	−72	32
	L Lingual Gyrus	12	3.369	6E-04	−3	−90	−17
Temporal	L Middle Temporal Gyrus	27	3.724	2E-04	−48	−68	12
	R Middle Temporal Gyrus	71	3.566	3E-04	48	−70	12
	L Fusiform Gyrus	137	3.437	4E-04	−38	−59	−16
	R Fusiform Gyrus	469	4.154	4E-05	46	−59	−16
	R Parahippocampal Gyrus	183	3.910	9E-05	20	−36	−1
	L Hippocampus	36	3.385	5E-04	−26	−35	0
Subcortical	L Pulvinar Thalamus	53	3.570	3E-04	−4	−30	−3
	R Caudate	18	3.823	1E-04	20	−14	20
Cerebellum	R Cerebellum Lobule VI	94	3.594	3E-04	31	−44	−21
	Vermis VI	55	3.362	6E-04	−1	−63	−22
	Vermis VII	21	3.248	8E-04	3	−78	−27

k, cluster size; peak T, t-value at the peak voxel; peak p, p-value at the peak voxel; x, y, z, MNI coordinates of the peak voxel.

In contrast, in the control subjects several regions in frontal cortex, occipital cortex, cerebellum, and putamen showed decreased activation for dual tasking from session 2 to session 4 (Table 5), however no activation increase from session 2 to session 4 was found. The opposing direction of these changes versus those seen in the HDBR subjects supports the specificity of HDBR effects resulting from the intervention itself rather than practice effects.

**Table 5:** **Regions showing decreases from session 2 to session 4 in activation for dual tasking in the control subjects**.

**Region**		***k***	**Peak *T***	**Peak *p***	***x,y,z* (mm)**		
Frontal	R Superior Frontal Gyrus	30	4.173	1E-04	22	0	52
	L Supplementary Motor Cortex	31	4.064	1E-04	−4	8	42
	R Supplementary Motor Cortex	14	3.667	4E-04	12	4	50
Occipital	L Inferior Occipital Gyrus	25	4.148	1E-04	−42	−84	−8
	R Fusiform Gyrus	24	3.979	2E-04	42	−66	−16
	L Fusiform Gyrus	208	3.842	3E-04	−46	−59	−20
	L Middle Occipital Gyrus	62	3.717	4E-04	−30	−78	22
	R Middle Occipital Gyrus	37	3.555	6E-04	30	−76	28
Subcortical	L Putamen	10	3.712	4E-04	−22	22	−4
Cerebellum	L Cerebellum Lobule VI	393	4.433	5E-05	−33	−74	−19

k, cluster size; peak T, t-value at the peak voxel; peak p, p-value at the peak voxel; x, y, z, MNI coordinates of the peak voxel.

#### Brain-behavioral correlation for tapping under dual task condition

The pre-to-post HDBR difference in tapping reaction time (dual task condition) was positively associated with pre-to-post HDBR changes of brain activation in a few clusters in the frontal, parietal, temporal, and occipital lobes (Table 6), i.e., greater slowing of reaction time was associated with greater activation increases in these regions. The opposite relationship was found in three clusters within the brain stem and cerebellum (Table 6).

**Table 6:** **Regions showing associations between pre-to-post HDBR differences in tapping reaction time and brain activation for dual tasking**.

	**Region**		***k***	**Peak *T***	**Peak *p***	***x,y,z* (mm)**		
Positive correlation	Frontal	R Middle Frontal Gyrus	44	5.386	8E-05	16	36	32
		R Middle Frontal Gyrus	28	4.585	3E-04	40	12	38
	Parietal	R Precuneus	82	5.373	8E-05	10	−54	38
	Temporal	L Superior Temporal Gyrus	24	4.869	2E-04	−58	−14	−2
	Occipital	R Inferior Occipital Gyrus	43	4.396	4E-04	19	−93	−8
Negative correlation	Brain stem	Nuclei Gracile	248	5.089	1E-04	0	−41	−58
		Cuneate Nuclei	28	4.390	4E-04	4	−50	−60
	Cerebellum	Cerebellar Tonsil	17	4.317	5E-04	−7	−34	−43

k, cluster size; peak T, t-value at the peak voxel; peak p, p-value at the peak voxel; x, y, z, MNI coordinates of the peak voxel.

#### Brain activation change for dual-task cost on tapping

**Figure 8:**
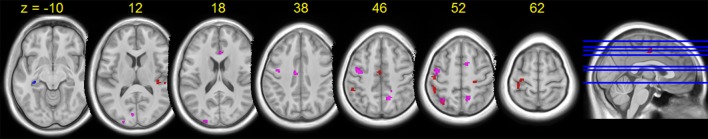
**Group T-statistic maps for the regions showing HDBR-related changes in dual tasking cost of brain activation, thresholded at abs (T) > 3.218**. Red: HDBR-related cumulative increases; blue: HDBR-related cumulative decreases; violet: HDBR-related immediate increases.

HDBR-related cumulative increases in dual-task cost of brain activation was observed in frontal, parietal, temporal, and cerebellar areas (Figure 8, Table 7). A cluster in the left hippocampus showed cumulative decreases during HDBR.

**Table 7:** **Regions showing HDBR-related cumulative increases in dual-task costs of brain activation**.

**Region**		***k***	**Peak *T***	**Peak *p***	***x,y,z* (mm)**		
Parietal	L Inferior Parietal Gyrus	87	4.760	4E-06	−50	−40	50
	L Postcentral Gyrus	87	3.792	1E-04	−36	−34	62
	L Superior Parietal Gyrus	41	3.722	2E-04	−32	−62	52
	L Postcentral Gyrus	21	3.654	2E-04	−48	−20	52
Frontal	R Precentral Gyrus	24	3.565	3E-04	24	−26	54
Cingulate	L Middle Cingulate Gyrus	47	3.507	4E-04	−4	−10	44
Temporal	R Middle Temporal Gyrus	176	3.540	3E-04	49	−44	0
	R Superior Temporal Gyrus	10	3.323	7E-04	50	−30	10
Insula	R Insula	22	3.497	4E-04	38	−26	14
Cerebellum	R Cerebellum Lobules IV & V	11	3.226	9E-04	19	−45	−27

k, cluster size; peak T, t-value at the peak voxel; peak p, p-value at the peak voxel; x, y, z, MNI coordinates of the peak voxel.

In contrast to the cumulative change regions, regions with HDBR-related immediate increase in dual-task cost exhibited a more frontal distribution. In addition to parietal, occipital, and temporal activation, several clusters were observed in frontal and cingulate cortices (Figure 8, Table 8). No immediate decrease was observed during HDBR.

**Table 8:** **Regions showing HDBR-related immediate increases in dual-task costs of brain activation**.

**Region**		***k***	**Peak *T***	**Peak *p***	***x,y,z* (mm)**		
Frontal	L Precentral Gyrus	192	3.984	7E-05	−40	−8	46
	R Supplementary Motor Cortex	41	3.963	8E-05	12	4	54
	R Middle Frontal Gyrus	17	3.509	4E-04	26	−6	42
	L Middle Frontal Gyrus	17	3.426	5E-04	−44	16	24
	L Precentral Gyrus	15	3.364	6E-04	−42	4	32
Parietal	R Precuneus	106	3.963	8E-05	14	−56	46
	L Superior Parietal Lobule	73	3.832	1E-04	−32	−60	52
	R Precuneus	10	3.486	4E-04	20	−44	44
Cingulate	R Anterior Cingulate Gyrus	35	3.842	1E-04	4	24	20
	L Middle Cingulate Gyrus	34	3.773	1E-04	−10	−12	38
	R Middle Cingulate Gyrus	20	3.529	3E-04	6	14	32
Occipital	L Middle Occipital Gyrus	40	3.433	5E-04	−22	−96	16
	L Cuneus	19	3.350	6E-04	−6	−84	12
Temporal	R Middle Temporal Gyrus	10	3.439	4E-04	60	−58	8

k, cluster size; peak T, t-value at the peak voxel; peak p, p-value at the peak voxel; x, y, z, MNI coordinates of the peak voxel.

For the control group, no increase in the dual-task cost for brain activation was found from session 2 to session 4. One cluster within the left cerebellum (crus I, 98 voxels) exhibited decreased dual-task cost of brain activation from session 2 to session 4 (peak *T* = 3.792). This cluster did not overlap with the brain areas that showed HDBR-related change in dual-task cost of brain activation.

#### Brain-behavioral correlation for dual-task cost of tapping

We observed a positive correlation between pre-to-post HDBR changes in dual-task cost on tapping reaction time and dual-task cost on brain activation in a distributed network, involving frontal, parietal, occipital, temporal, insula, and cerebellum areas (Table 9).

**Table 9:** **Regions showing positive correlations between pre-to-post HDBR changes in dual-task cost of tapping reaction time and dual-task cost of brain activation**.

**Region**		***k***	**Peak *T***	**Peak *p***	***x,y,z* (mm)**		
Frontal	R Superior Frontal Gyrus	113	5.383	8E-05	2	28	50
	R Superior Frontal Gyrus	28	5.038	1E-04	12	14	58
	R Inferior Frontal Gyrus	40	5.102	1E-04	44	10	16
	L Operculum	12	4.935	2E-04	−36	−8	22
Parietal	L Operculum	149	9.806	2E-07	−44	−18	26
	R Postcentral Gyrus	129	5.811	4E-05	34	−20	36
	R Inferior Parietal Lobule	24	5.059	1E-04	36	−36	42
	R Precuneus	10	4.308	5E-04	20	−46	36
	L Angular Gyrus	32	4.732	2E-04	−34	−52	26
Cingulate	L Middle Cingulate Gyrus	297	7.641	3E-06	−18	−14	46
	L Anterior Cingulate Gyrus	51	5.097	1E-04	−6	16	26
	R Middle Cingulate Gyrus	26	4.208	6E-04	18	10	36
	R Middle Cingulate Gyrus	42	10.446	1E-07	16	−38	40
Occipital	R Lingual Gyrus	11	4.237	6E-04	30	−63	6
	R Cuneus	14	4.268	5E-04	22	−72	26
	R Lingual Gyrus	43	4.817	2E-04	28	−51	−1
Temporal	R Middle Temporal Gyrus	512	6.308	2E-05	41	−45	0
	R Middle Temporal Gyrus	228	5.595	6E-05	50	−63	0
	L Middle Temporal Gyrus	476	5.757	5E-05	−51	−70	3
Insula	L Insula	26	4.462	4E-04	−30	16	10
Cerebellum	R Cerebellum Crus I	60	5.275	1E-04	44	−60	−29
	L Cerebellum IX	14	4.119	7E-04	−11	−54	−42

k, cluster size; peak T, t-value at the peak voxel; peak p, p-value at the peak voxel; x,y,z, MNI coordinates of the peak voxel.

## Discussion

The current study investigated the effect of long-term HDBR on dual task performance and the underlying brain activation. The frontal, parietal, cingulate, and temporal cortices exhibited increased activation for dual task execution during HDBR, which then recovered post HDBR, suggesting reduced neural efficiency in a spaceflight analog environment.

We observed activation in the frontal, parietal, and cingulate cortices during dual tasking at the pre-HDBR baseline test, consistent with the existing literature (D'Esposito et al., 1995; Szameitat et al., 2002; Marois et al., 2006). Immediate and cumulative HDBR-related increases of brain activation for dual tasking revealed that more neural resources were required for dual tasking during HDBR. These findings suggest that HDBR may result in lower efficiency of neural processing, which then recovered after HDBR. Bock attributes increased dual task costs inflight to the resource demands of adapting to microgravity (Bock et al., 2010); adaptation to the bed rest environment here may similarly limit resource availability. This change could result from physiological, perceptual, and psychological effects of bed rest. First, when a subject is in a head-down tilt supine position for a sustained period, the intravascular and extravascular fluids are shifted toward the head and upper body. Reports of changes in gray matter volume with HDBR suggest that fluid shifts and the brain settles into a new position toward the back of the skull (Roberts et al., 2010; Rao et al., 2014; Koppelmans et al., under review). The axial body unloading and potential sensory reweighing that occur during HDBR could tax the available resources. Both HDBR and spaceflight environments challenge the brain to adaptively change and this adaptive alteration may impact dual tasking performance. Second, participants exhibit changes in sensorimotor performance from pre to post HDBR which suggest sensory reweighting and adaptive neuroplasticity (Koppelmans et al., 2015). The resource demands of this process may limit dual tasking abilities. Third, the stress of being in an altered and somewhat isolated environment may also influence subjects' performance and corresponding brain activation patterns. Future studies could directly measure fluid shifts or changing cortisol levels with HDBR to determine how these relate to the increased neural dual task costs we observed here.

The HDBR-associated lower neural efficiency for dual tasking parallels findings from electroencephalographic (EEG) spaceflight studies. Cheron et al. recorded EEG activity during visual processing, and reported decreased event-related synchronization in the theta-alpha oscillations during spaceflight (Cheron et al., 2014). As the EEG alpha component amplitude is negatively associated with BOLD signals (Feige et al., 2005), the reduced alpha power for visual processing would be expected to link with increased BOLD activation (Cheron et al., 2014), and decreased neural efficiency during spaceflight. Cheron et al. also measured spontaneous EEG (while participants were not performing a task) and found that the alpha power increased in the eyes-closed state but did not significantly change in the eyes-open state during spaceflight (Cheron et al., 2006), ruling out the existence of general reductions of alpha power in microgravity. The reduction of neural efficiency for dual tasking in our current study thus supports Cheron's previous findings that neural efficiency for task processing decreases during spaceflight.

When comparing dual task performance of HDBR subjects during HDBR with the performance of control subjects over a similar time course, we observed lower accuracy of counting in the HDBR group than in the control group, but no group difference in the other cognitive scores such as tapping latency and accuracy. The relative retention of dual tasking performance reflects greater frontal and parietal recruitment to maintain the same performance level during HDBR.

We found that dual task processing is more susceptible to HDBR than single task performance: Behaviorally, the HDBR-associated group difference (counting accuracy) was only evident under the dual task condition rather than the single task one. Moreover, the immediate HDBR-related brain activation increase was found for dual tasking but not for single tasking. This is similar to what has been observed in terms of behavior inflight; manual sensorimotor impairments are more evident under dual tasking conditions (Manzey et al., 1995, 1998; Bock et al., 2010). Thus our findings suggest that the spaceflight-induced declines in dual tasking could at least partly be attributed to cephalic fluid shifts, axial body unloading, and sensorimotor adaptation, which were at least partially simulated by HDBR. Spaceflight-specific factors other than those mentioned might of course also contribute to spaceflight-induced declines in dual tasking.

The effect of HDBR on brain activation takes place very quickly, even within 7 days after the onset of HDBR, as expressed by the immediate HDBR-related change in activation for dual tasking. As illustrated in Figure 7, the immediate HDBR-related activation increase for dual tasking was mainly distributed in the frontal, parietal, and cingulate regions—the brain areas that are important for dual task processing. On the other hand, the cumulative HDBR-related activation change for dual tasking was focused at the parietal area especially in the precuneus. In referring to the baseline deactivation regions (Figure 5), the cumulative HDBR-related activation change in Figure 7 is more likely to be due to HDBR-related decreases in deactivation, rather than increases in activation. It has been reported that the precuneus area exhibits gray matter volumetric increases with HDBR (Koppelmans et al., under review), thus it is possible that HDBR-related changes in functional activation could partly be due to HDBR-related alterations in cortical structure. Our results of the immediate HDBR-associated activation change revealed that the HDBR effects occurred as soon as 7 days in HDBR, however, determining the onset of neural activation change would require further investigation with more frequent measurements. The temporal dynamics of HDBR-related behavioral and neural changes would be more precisely quantified if the behavioral and fMRI measures were collected more frequently, e.g., daily.

The brain regions showing activation changes from session 2 to session 4 in the control subjects did not overlap with the regions showing HDBR-related changes, implying that the latter were not merely due to practice effects. Moreover, the brain activations for dual task and dual-task cost prevailingly increased in certain areas with HDBR, whereas in control participants brain activation decreased with repeated measurements. The opposing directions of these changes support the specificity of the HDBR effects and their mechanism as due to the HDBR intervention but not practice. Furthermore, according to previous reports (Erickson et al., 2007; Wu et al., 2013), if practice did play a role in the functional activation change, then such a practice-related change should be reductions in activation. The observed activation reduction in control subjects likely reflects practice-induced improvements in neural efficiency, which is consistent with previous literature.

The training-induced reduction in brain activation may represent improvement in the efficiency of dual task processing. Previous studies on dual task training have reported an association between brain activation decreases and performance improvements for dual tasking (Erickson et al., 2007; Wu et al., 2013). Thus, the reduction in brain activation could indicate increased neural efficiency, i.e., less neural resources are needed to complete the task (Wu et al., 2013). In light of this literature, subjects in the current study who obtained greater behavioral improvement from repeated training would have a tendency to exhibit greater enhancement in neural efficiency, and then show more reduction in brain activation. Such an inference was supported by our results. We observed a positive correlation between pre-to-post HDBR change in dual-task cost for tapping reaction time and pre-to-post HDBR activation change in frontal, parietal, temporal, and cingulate areas, which verified our hypothesis. This brain-behavior association could be used to predict behavioral change from pre-to-post HDBR changes in the activation of certain brain areas, i.e., subjects with more pre-to-post HDBR increase in brain activation are likely to exhibit larger increase in dual-task cost for tapping reaction time.

The current study investigated the effects of HDBR on brain activation for dual tasking, and provided insight into the effects of spaceflight on neural efficiency. However, generalizing the HDBR findings to spaceflight should be approached with caution. First, as an analog environment, HDBR only mimics particular characteristics of spaceflight such as foot sole unloading, fluid shifts, and sensorimotor adaptation, but not all the features of spaceflight. Second, as revealed by EEG studies, microgravity effects on brain activation are task dependent (Cheron et al., 2006, 2014). Thus the factors influencing neural activation during spaceflight could differ from those reported here. Even in the microgravity environment itself behavior can be context specific. For example, during spaceflight, up-down turning asymmetry was immediately reduced when astronauts were free-floating as compared to tethered (De Saedeleer et al., 2013). The current study is also limited by the inequality in the number of sessions for the HDBR and control groups. Future studies using equal numbers of sessions and similar timelines for both groups would provide more precise information about the temporal dynamics of HDBR effects on dual-tasking behavior. Furthermore, in the current study, only male subjects were included in the HDBR group, as they were serving as controls for a separate investigator's study on testosterone to reduce HDBR decrements. Future studies involving both males and females would provide more comprehensive information and could better generalize to the broader astronaut population.

## Conclusion

The current study investigated the effect of 70-day HDBR on dual task performance and the underlying brain activation. Impairments of dual task performance were found in HDBR subjects compared to control subjects. Furthermore, we observed increased activations for dual tasking during HDBR in a widely distributed network involving frontal, parietal, occipital, temporal, cingulate, cerebellar, and subcortical regions. By comparing with activation change in control subjects, we demonstrated that the HDBR-associated brain activation increase was due to the HDBR intervention itself, rather than repeated testing. These results demonstrate lower neural efficiency for dual task processing during HDBR. As a microgravity analog, HDBR has successfully simulated a variety of microgravity exposure effects on human physiology and cognition. Therefore, we conjecture similar behavioral and neural changes occur during spaceflight to those during HDBR. This hypothesis warrants further investigation based on data from astronauts before, during and after their space missions.

## Author contributions

PY analyzed data and wrote the manuscript, VK analyzed data and wrote the manuscript, PR designed the study and wrote the manuscript, YD collected data and wrote the manuscript, NG collected data and wrote the manuscript, SW, RR, IK, JB, AM, and RS designed the study and wrote the manuscript.

## Funding

This work is supported by grants from the National Space Biomedical Research Institute (NASA NCC 9-58, MA02701, and PF04101), from the National Aeronautics and Space Administration (NASA; NNX11AR02G) and NASA Flight Analogs Project, and the National Institutes of Health, and National Center for Advancing Translational Sciences, 1UL1RR029876-01.

### Conflict of interest statement

The authors declare that the research was conducted in the absence of any commercial or financial relationships that could be construed as a potential conflict of interest.
